# Arsenic compounds activate MAPK and inhibit Akt pathways to induce apoptosis in MA‐10 mouse Leydig tumor cells

**DOI:** 10.1002/cam4.5068

**Published:** 2022-08-24

**Authors:** Wei‐Sheng Juan, Yi‐Fen Mu, Chia‐Yih Wang, Edmund‐Cheung So, Yi‐Ping Lee, Sheng‐Che Lin, Bu‐Miin Huang

**Affiliations:** ^1^ Department of Neurosurgery, An Nan Hospital China Medical University Tainan City Taiwan; ^2^ Department of Cell Biology and Anatomy, College of Medicine National Cheng Kung University Tainan Taiwan; ^3^ Department of Anesthesia & Medical Research, An Nan Hospital China Medical University Tainan City Taiwan; ^4^ Department of Plastic Surgical, An Nan Hospital China Medical University Tainan City Taiwan; ^5^ Department of Medical Research, China Medical University Hospital China Medical University Taichung Taiwan, Republic of China

**Keywords:** Akt, dimethylarsenic acid, Leydig tumor cell, MAPK, ROS, sodium arsenite

## Abstract

Arsenic compounds have been applied treating acute promyelocytic 1eukemia and solid tumors with brief mechanism investigations. In fact, we have demonstrated that sodium arsenite plus dimethylarsenic acid could activate apoptosis in MA‐10 mouse Leydig tumor cells by inducing caspase pathways. However, detail underlying mechanisms how caspase cascade is regulated remains elusive. Therefore, the apoptotic mechanism of sodium arsenite plus dimethylarsenic acid were examined in MA‐10 cells in this study. Our results reveal that Fas/FasL protein expressions were stimulated by sodium arsenite plus dimethylarsenic acid in MA‐10 cells. In addition, *reactive oxygen species* (ROS) generation, cytochrome C release, Bid truncation, and Bax translocation were induced in MA‐10 cells by arsenic compounds. Moreover, activation of p38, JNK and ERK1/2, MAPK pathways was stimulated while Akt phosphorylated levels and Akt expression were decreased by sodium arsenite plus dimethylarsenic in MA‐10 cells. In conclusion, sodium arsenite and dimethylarsenic acid did activate MAPK pathway plus ROS generation, but suppress Akt pathway, to modulate caspase pathway and then induce MA‐10 cell apoptosis.

## Graphical abstract

Sodium arsenite and dimethylarsenic acid could activate MAPK pathway plus ROS generation with the suppression of Akt pathway to modulate caspase pathway and then induce MA‐10 cell apoptosis.

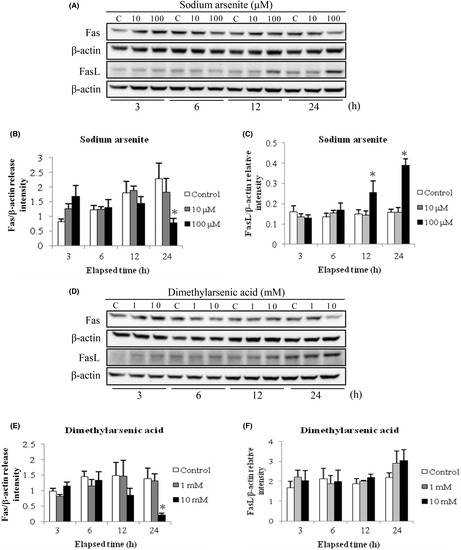

## 1 INTRODUCTION

Arsenic is a common environmental metalloid with inorganic arsenic compounds, such as arsenic trioxide (As_2_O_3_) and realgar (As_2_S_2_), and organic arsenic compounds, such as dimethylarsenic acid ([CH3]_2_AsO_2_H; DMA).^1^, ^2^ Some studies have demonstrated arsenic trioxide may induce cell death and apoptosis in prostate cancer, ovarian cancer, TM4 Sertoli tumor cells, and oral cancers.^3^, ^4^, ^5^, ^6^, ^7^ It has been shown dimethylarsenic acid and phenylarsonous acid had in vitro plus clinical investigations related to cancer therapy.^8^ In fact, arsenic trioxide has been approved for an anti‐tumor drug to treat acute promyelocytic leukemia.^9^, ^10^ Consequently, inorganic plus organic arsenic compounds could possibly be used to treat testicular tumors.

It is well known that the Fas/FasL will bind to death receptor to activate caspase‐8 and the stress signals will trigger mitochondria to release cytochrome C inducing the formation of apoptosome to activate caspase‐9,^11^, ^12^, ^13^ which will eventually cause activation of *Poly (ADP‐ribose) polymerase* (PARP) and caspase‐3 inducing cell apoptotic death.^14^ In addition, Bcl‐2 family proteins play different physiological roles for mitochondrial integrity, including Bcl‐xL and Bcl‐2 multidomain antiapoptotic, Bak and Bax multidomain proapoptotic, and Bmf and Bid BH3‐only proapoptotic proteins.^12^, ^15^ These proteins could negatively or positively regulate the permeability of mitochondria and the efflux of proteins related to apoptosis.^16^, ^17^ It is shown that arsenite trioxide could upregulate BH3‐only proapoptotic proteins and downregulate antiapoptotic proteins in myeloma.^18^ However, Fas/FasL extrinsic pathway similarly participated in apoptosis in arsenic‐induced keratinocytes.^19^ Accordingly, the mechanisms of arsenic‐induced apoptosis among various tumor cell types are complicated and remain elusive.

Mitogen‐activated protein kinase (MAPK) pathways are important signaling in response to external stimuli, including p38, JNK and ERK1/2 proteins.^20^ It is known the activation of MAPK could influence downstream signal transduction and physiological behaviors, including cell mitosis, proliferation and apoptosis.^21^, ^22^ It has been found that arsenic‐induced apoptosis could be mediated by stimulation of MAPK in mesothelioma cells^23^ and cervical cancer cells.^24^ Akt is another essential kinase for cell growth by activation of survival signaling and attenuation of apoptosis pathway through phosphorylation of caspase‐9,^25^ and it is illustrated that dysregulation of Akt pathway is one of the factors in testicular stromal tumor formation.^26^ In addition, reactive oxygen species (ROS) display various physiological and pathological roles according to the level of intracellular ROS.^27^ Physiologically, ROS serves as signaling molecules to stimulate cell proliferation through modulating several signaling, such as MAPK pathways. However, excessive ROS generation could induce cell apoptosis because of the irreversible damages of DNA and proteins.^28^ It is shown that sodium arsenite could induce ROS production in human vascular smooth muscle^29^ and U937 leukemia cells.^30^ Moreover, it was found that sodium arsenite could induce apoptosis through oxidative stress in rat testes.^31^

We have illustrated sodium arsenite plus dimethylarsenic acid could stimulate both extrinsic death receptor and intrinsic mitochondrial caspase cascades to induce apoptosis in MA‐10 mouse Leydig tumor cells.^5^ However, the detail underlying mechanisms how caspase cascade is regulated still remains unclear. To reveal the uncertain mechanisms of arsenic‐induced cell apoptosis in Leydig cell tumors, sodium arsenite and dimethylarsenic acid were used to treat MA‐10 cells. Then, activations of caspase, Akt and MAPK signalings plus ROS changes were investigated. The mechanism findings in the present study would be helpful to provide effective chemotherapy strategies upon Leydig and reproductive‐related tumors.

## 2 MATERIALS

### 2.1 Chemicals

Dimethylarsenic acid, RNase A, Waymouth MB 752/1 medium, ethylene diamine tetraacetic acid, propidium iodide, ethylene glycol tetraacetic acid, Folin & Ciocalteu's phenol reagent, 30% acrylamide/Bis‐acrylamide solution, 2′,7′‐dichlorofluorescin diacetate and monoclonal antibody against β‐actin (#A5441; 1/8000) were obtained from Sigma‐Aldrich; Merck KGaA (Darmstadt, Germany). Sodium arsenite was bought from Fluka (St. Gallen, Switzerland). Gentamycin sulfate was bought from AG Scientific Inc. (San Diego, CA, United States). Trypsin–EDTA and fetal bovine serum were acquired from Gibco; Thermo Fisher Scientific, Inc. (Waltham, MA, USA). The 4‐(2‐hydroxyethyl)‐1‐piperazineethanesulfonic acid, sodium chloride, and Tris base potassium chloride were bought from J.T. Baker (Phillipsburg, NJ, United States). Tissue culture grade sodium bicarbonate, potassium dihydrogen phosphate, and disodium hydrogen phosphate were obtained from Riedel‐deHaen (Seelze, Germany). Sucrose was bought from Panreac (Barcelona, Spain). Dimethyl sulfoxide, Hydrochloric acid, sodium dodecyl sulfate, and Tween 20 were purchased from Merck (Darmstadt, Germany). Donkey anti‐mouse IgG conjugated with horseradish peroxidase (HRP) and donkey anti‐rabbit IgG conjugated with HRP were obtained from PerkinElmer (Boston, MA, United States). Polyclonal antibodies against cytochrome C (#4272; 1/2000), COX IV (#4844; 1/2000), ERK1/2 (#9102; 1/4000), phospho‐ERK1/2 (#9101; 1/4000), JNK (#9252; 1/1000), phospho‐JNK (#9251; 1/4000), p38 (#9212; 1/4000), phospho‐p38 (#9215; 1/1000), Akt (#9272; 1/1000), phospho‐Akt (#9271; 1/4000), and Bax (#2772; 1/1000) were bought from Cell Signaling (Beverly, MA, United States). Polyclonal antibody for Bid (#2002; 1/1000) and Fas (#4233; 1/1000) were obtained from Santa Cruz (Santa Cruz, CA, United States). Polyclonal antibody against Fas ligand (ab89292; 1/2000) was bought from Abcam (Cambridge, UK). Enhanced chemiluminescence detection kit was obtained from Millipore (Billerica, MA, United States).

### 2.2 Cells

MA‐10 mouse Leydig tumor cell line was from Dr. Mario Ascoli (University of Iowa, Iowa City, IA, United States), which was maintained in standard method. MA‐10 cells were incubated in a humidified atmosphere at 37°C containing 5% CO_2_ and 95% air in Waymouth medium with 10% fetal bovine serum.^32^, ^33^, ^34^, ^35^

### 2.3 Protein extraction and Western blotting analysis

The 6 × 10^6^ MA‐10 cells were plated in 60‐mm dish, and the treated cells were lysed with 100 μl lysis buffer (150 mM NaCl, 20 mM Tris pH 7.5, 1 mM EGTA, 1 mM sodium orthovanadate, 2.5 mM sodium pyrophosphate, 1% Triton X‐100, and 1 mM EDTA). The cell lysates were collected with centrifugation at 12,000 X *g* at 4°C for 12 min. Supernatants contained total proteins were kept at −20°C. The concentration of protein was evaluated by Lowry assay.^36^ For western blot analysis, 30 μg total proteins were analyzed on 12% SDS‐PAGE gel and transferred to polyvinyldifluoride membranes, electrophoretically. Membranes were then incubated overnight at 4°C with 1° antibodies after 5% milk in Tris‐buffered saline containing 0.1% Tween 20 blocking solution for 1 h at 25°C. Following extensive washes and incubation with appropriate HRP‐conjugated 2° antibodies for 1 h, the blots were visualized by enhanced chemiluminescence detection kit with UVP EC3 BioImaging Systems (UVP, Upland, CA, United States).^37^ The quantificational analysis was performed by Image J program (NIH, Bethesda, MD, USA).

### 2.4 Isolation of mitochondrial protein

The 1.7 × 10^7^ MA‐10 cells were plated in 100‐mm dishes. Treated cells were scraped down with 4 ml cold PBS and centrifuged with 3200 rpm at 4°C for 10 min, and the pellets were resuspened by isolation buffer (0.25 M sucrose, 10 mM Tris and 0.1 mM EDTA; pH 7.4). Cells were then homogenized with 1000 rpm for 22 strokes by motorized glass homogenizer. Homogenates were centrifuged for 30 min at 600 × *g*. The resultant supernatants were further centrifuged for 30 more min at 12,000 × *g*. Pellets as mitochondrial fractions were collected by lysis buffer (50 μl) and supernatants were collected as cytosolic fractions. Mitochondrial and cytosolic fractions (30 μg) were further investigated with western blotting.^38^

### 2.5 Intracellular ROS generation

Intracellular ROS production in MA‐10 cells was detected by 2′,7′‐dichlorofluorescin diacetate (DCFDA) through flow cytometry.^39^ The 4.5 × 10^6^ MA‐10 cells were plated in 60‐mm dishes. Treated cells were then stained with 0.25 μM DCFDA for 5 min prior to the end of treatment time. MA‐10 cells were harvested by trypsin digestion with centrifugation at 4°C under 1000 rpm for 10 min. After extensive wash by isoton II, stained cells were determined with *λ* = 488 nm excitation by 515 nm band pass filter for FITC detection with FACS Calibar flow cytometer (Becton‐Dickinson, Mountain View, CA, United States). Fluorescence intensity indicated ROS generation level in MA‐10 cells.

### 2.6 Statistic

Data were all illustrated as means ± standard error of the mean of 3 independent experiments. Statistical significance of differences between control and treated groups among different time points was analyzed by two‐way analysis of variance and then Least Significance Difference, which was accomplished by using GraphPad Prism version 6 (GraphPad Software, Inc., La Jolla, CA, United States). *p* < 0.05 indicates significant difference.

## 3 RESULTS

### 3.1 Arsenic compounds affected Fas and Fas ligand expressions in MA‐10 cell apoptosis

**FIGURE 1:**
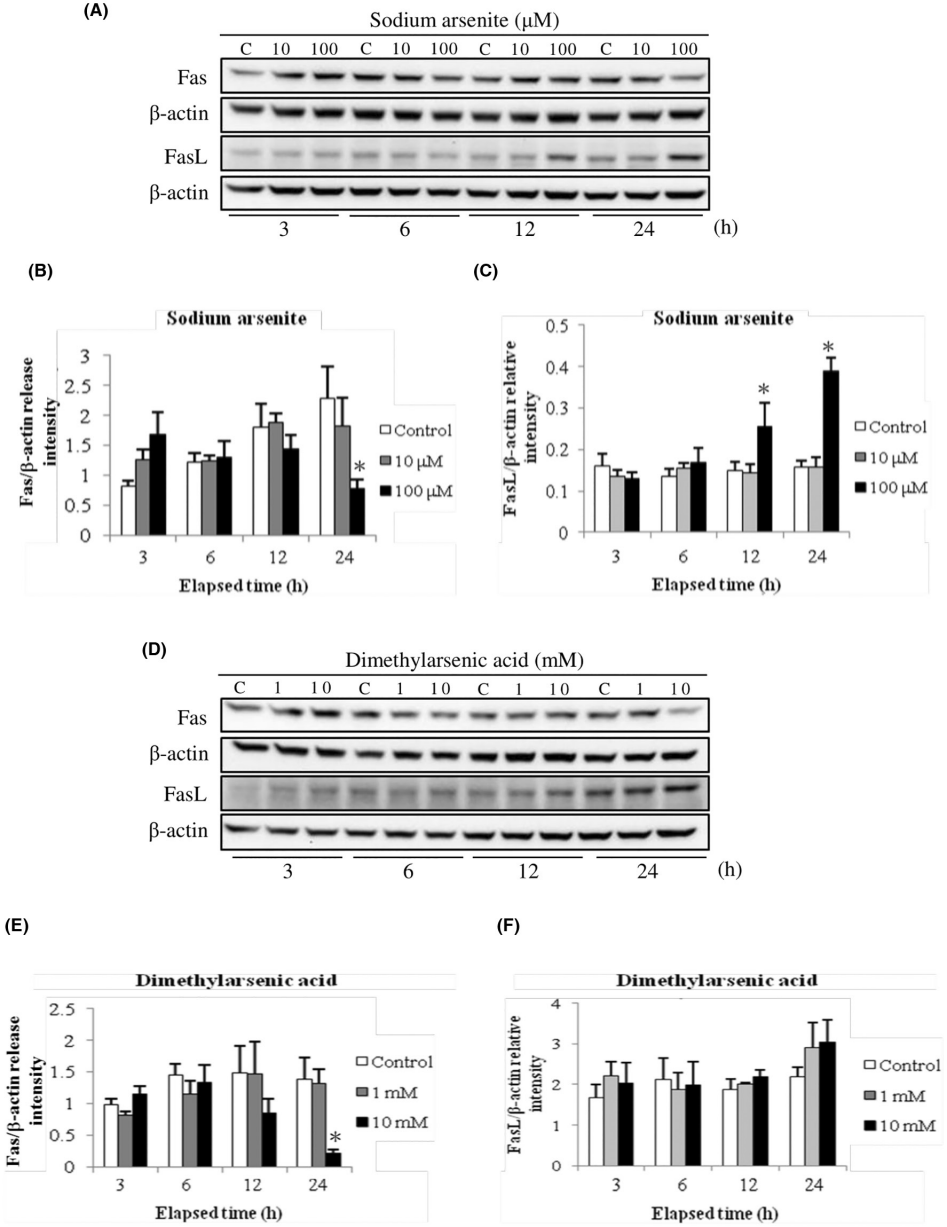
Sodium arsenite and dimethylarsenic acid regulated Fas and Fas ligand expressions in MA‐10 cells. MA‐10 cells were challenged with sodium arsenite (0, 10 and 100 μM) or dimethylarsenic acid (0, 1 and 10 mM) for 3, 6, 12 and 24 h, respectively. The expression of Fas (48 kDa) and FasL (31 kDa) was detected via western blotting assay. Represent immunoblots are from one experiment repeated at least 3 times (A for sodium arsenite treatment and D for dimethylarsenic acid treatment). The IOD of Fas (B for sodium arsenite treatment and E for dimethylarsenic acid treatment) and FasL (C for sodium arsenite treatment and F for dimethylarsenic acid treatment) was determined with β‐Actin (43 kDa) normalization for each lane, respectively. Results among B, C, E and F illustrate the mean ± SEM of 3 independent experiments. The * represents statistical difference as compared to control group, respectively (*p* < 0.05) (C = control in A and D).

Fas/FasL signaling is one of death receptor pathways which would trigger death signaling and caspase‐8 activation in cells.^40^ As caspase‐8 was induced by arsenic compounds in MA‐10 cell apoptosis,^5^ Fas and FasL expressions were investigated with western blot assay. Results showed that 100 μM sodium arsenite significantly stimulated FasL protein expression at 12 and 24 h (Figure 1A,C) (*p* < 0.05). Different from sodium arsenite treatment groups, dimethylarsenic acid slightly induced FasL protein expression at 24 h (Figure 1D,F). Meanwhile, the expression of Fas protein slightly increased after 3 h treatment with dimethylarsenic acid and sodium arsenite. However, Fas protein expression significantly decreased at 24 h treated with 100 μM sodium arsenite and 10 mM dimethylarsenic acid (Figure 1B,E) (*p* < 0.05). These results indicate Fas/FasL signaling could participate in sodium arsenite‐induced MA‐10 cell apoptosis, but Fas/FasL signaling could play a minor role in dimethylarsenic acid‐induced MA‐10 cell apoptosis.

### 3.2 Arsenic compounds affected cytochrome C release and Bcl‐2 family protein translocation in MA‐10 cell apoptosis

**FIGURE 2:**
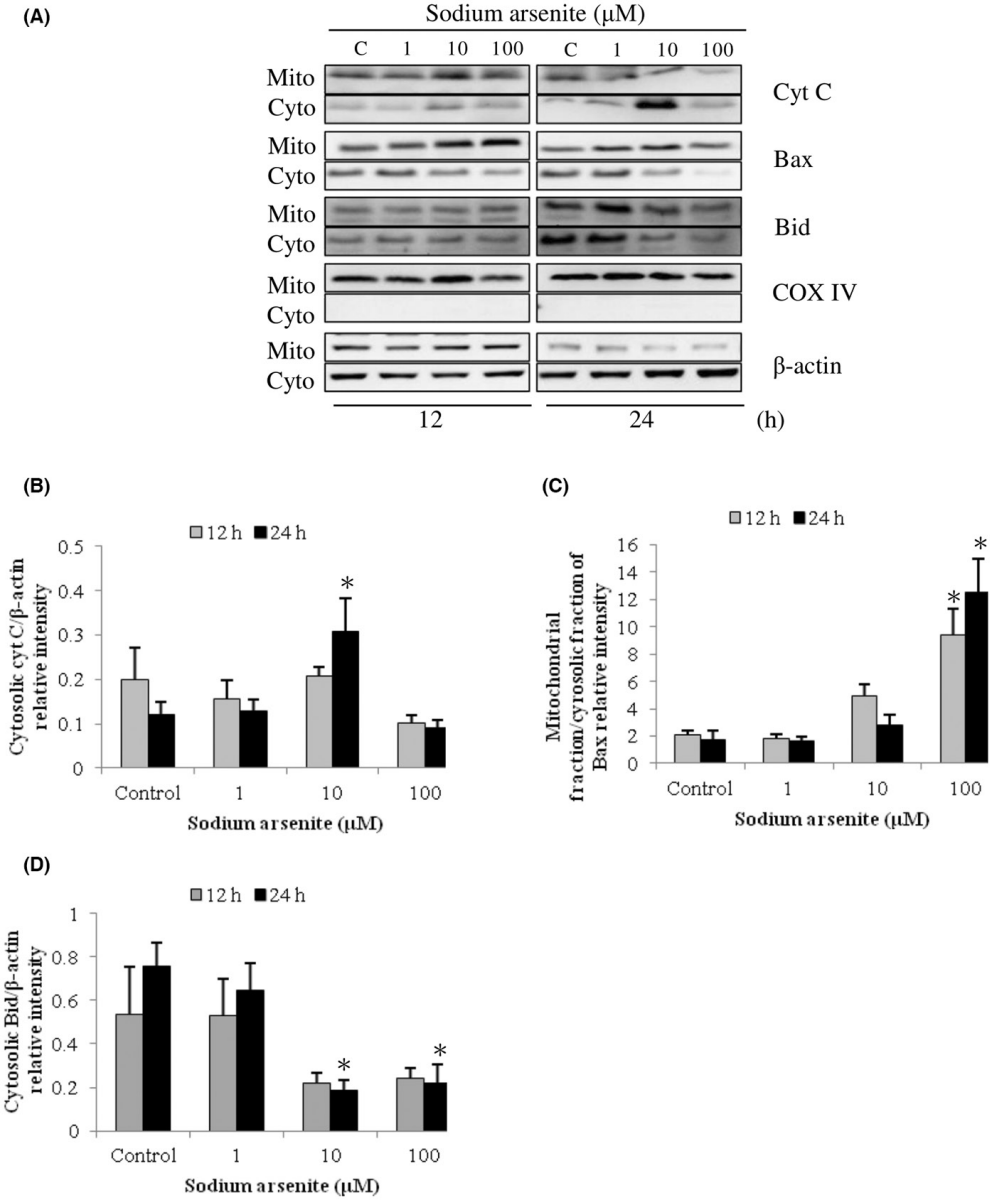
Dose effects of sodium arsenite on cytochrome C release and Bcl‐2 family protein translocation in MA‐10 cells. Cells were challenged with sodium arsenite (0, 1, 10 and 100 μM) for 12 and 24 h, respectively. Cytochrome C (14 kDa), Bid (22 kDa), and Bax (20 kDa) were, respectively, examined in mitochondrial (Mito) and cytosolic (Cyto) fractions via western blotting. Represent immunoblots are from one single experiment repeated at least 3 times (A). COX IV (17 kDa) and β‐Actin (43 kDa) were exploited as loading controls for cytosolic and mitochondrial fractions, correspondingly. IOD of cytochrome C (B), Bax (C), and Bid (D) was determined with loading control normalization for each lane. Results in B, C, and D illustrate the mean ± SEM of 3 independent experiments. The * represents statistical difference as compared to control group, respectively (*p* < 0.05).

**FIGURE 3:**
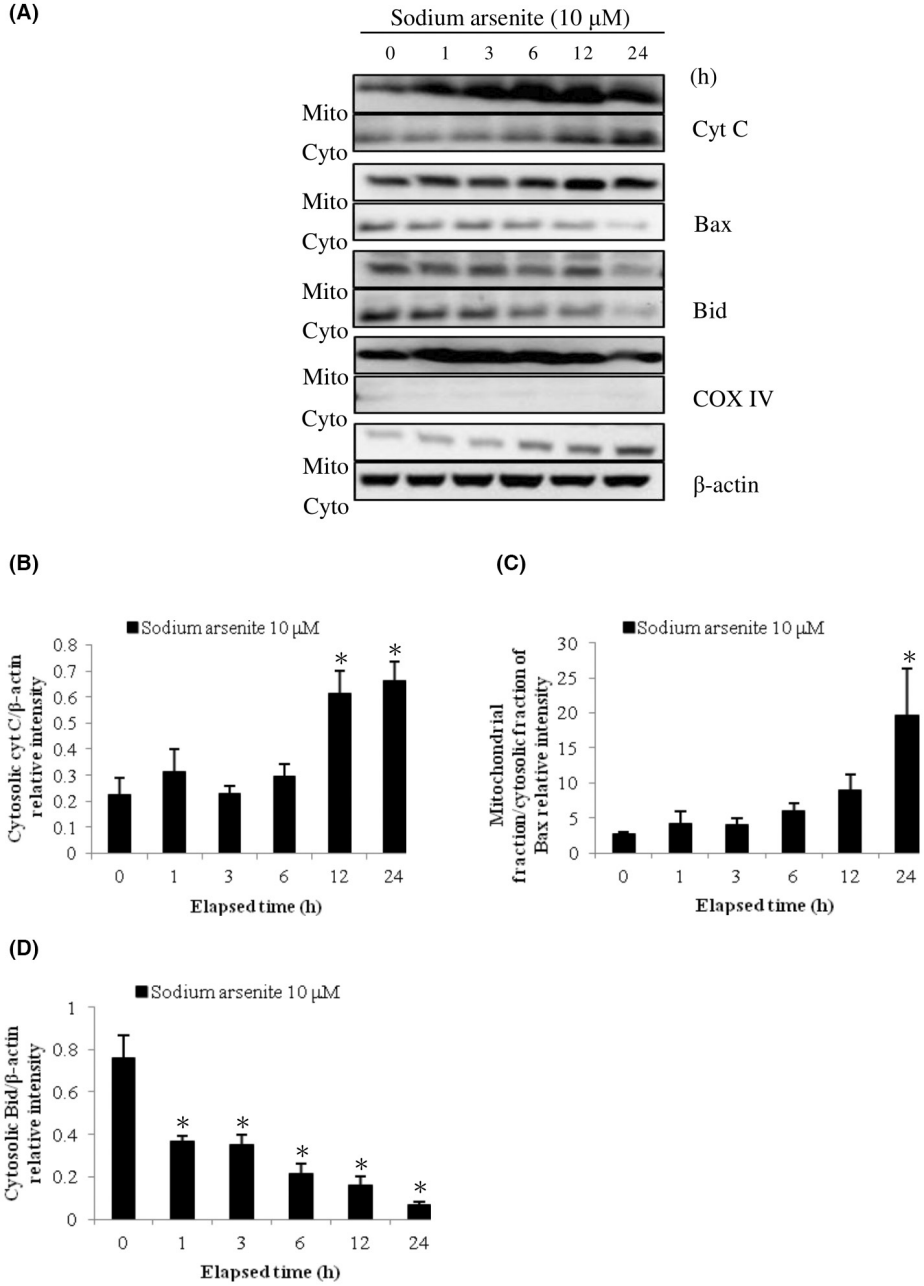
Temporal effects of sodium arsenite on cytochrome C release and Bcl‐2 family protein translocation in MA‐10 cells. Cells were challenged with 10 μM sodium arsenite for 0, 1, 3, 6, 12, and 24 h. Cytochrome C, Bax, and Bid were respectively examined in mitochondrial (Mito) and cytosolic (Cyto) fractions via western blotting. Represent immunoblots are from one single experiment repeated at least 3 times (A). COX IV (17 kDa) and β‐Actin (43 kDa) were exploited as loading controls for cytosolic and mitochondrial fractions, correspondingly. IOD of cytochrome C (B), Bax (C), and Bid (D) was determined with loading control normalization for each lane. Results in B, C, and D illustrate the mean ± SEM of 3 independent experiments. The * represents statistical difference as compared to control group, respectively (*p* < 0.05).

**FIGURE 4:**
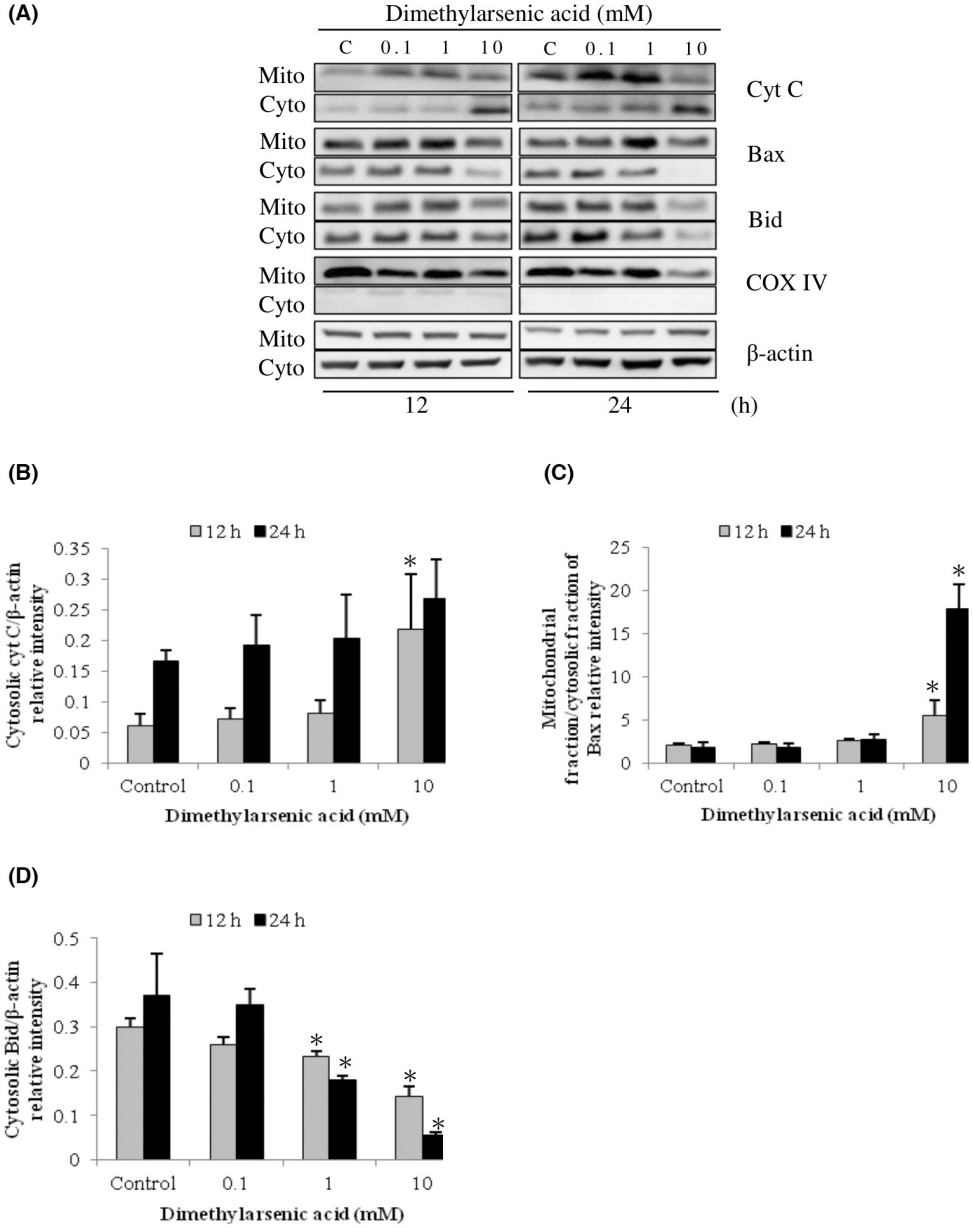
Dose effects of dimethylarsenic acid on cytochrome C release and Bcl‐2 family protein translocation in MA‐10 cells. Cells were challenged with dimethylarsenic acid (0, 0.1, 1 and 10 mM) for 12 and 24 h, respectively. Cytochrome C (14 kDa), Bid (22 kDa), and Bax (20 kDa) were respectively examined in mitochondrial (Mito) and cytosolic (Cyto) fractions via western blotting. Represent immunoblots are from one single experiment repeated at least 3 times (A). COX IV (17 kDa) and β‐Actin (43 kDa) were exploited as loading controls for cytosolic and mitochondrial fractions, correspondingly. IOD of cytochrome C (B), Bax (C), and Bid (D) was determined with loading control normalization for each lane. Results in B, C, and D illustrate the mean ± SEM of 3 independent experiments. The * represents statistical difference as compared to control group, respectively (*p* < 0.05).

**FIGURE 5:**
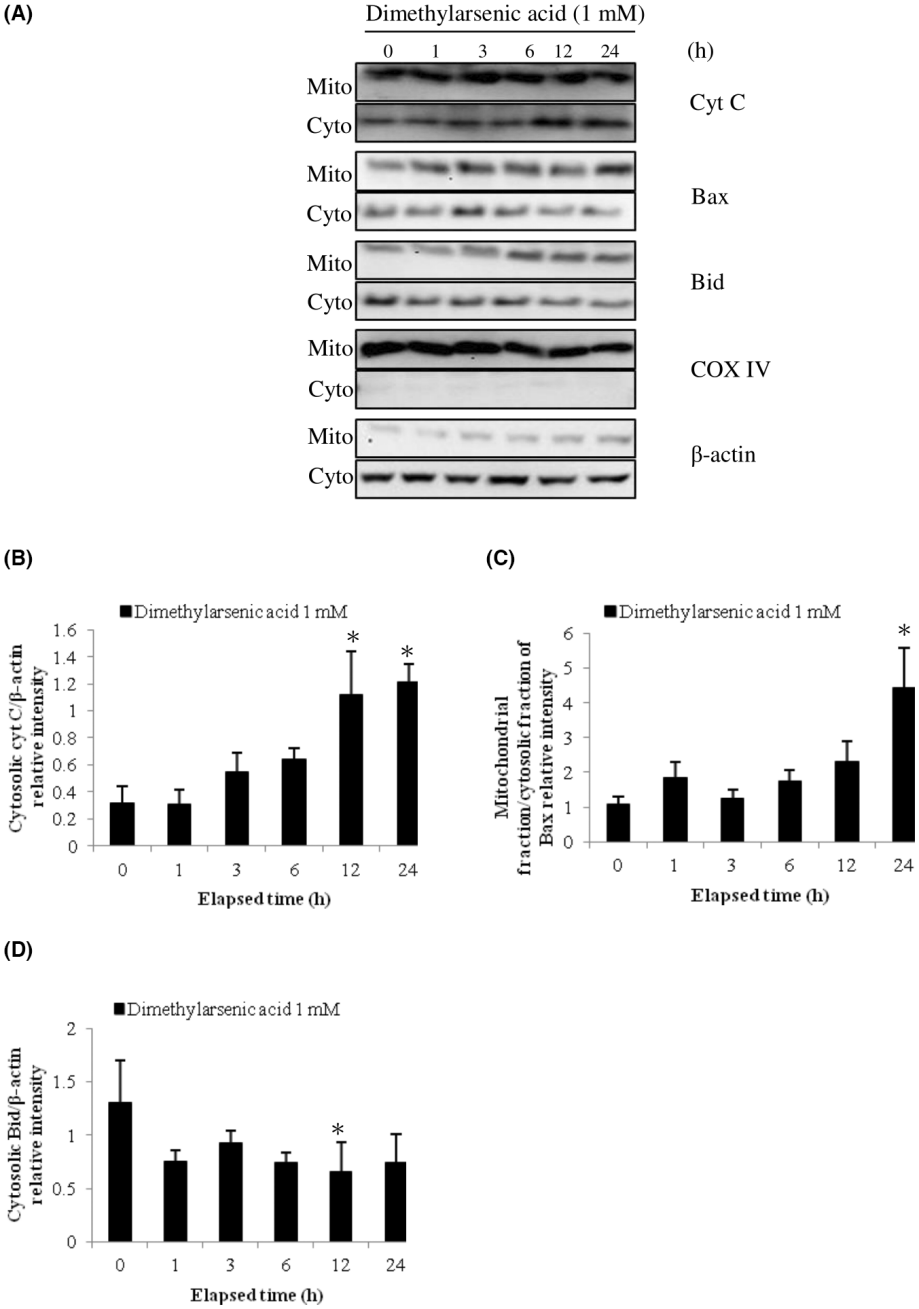
Temporal effects of dimethylarsenic acid on cytochrome C release and Bcl‐2 family protein translocation in MA‐10 cells. Cells were challenged with 1 mM dimethylarsenic acid for 0, 1, 3, 6, 12, and 24 h. Cytochrome C, Bax, and Bid were, respectively, examined in mitochondrial (Mito) and cytosolic (Cyto) fractions via western blotting. Represent immunoblots are from one single experiment repeated at least 3 times (A). COX IV (17 kDa) and β‐Actin (43 kDa) were exploited as loading controls for cytosolic and mitochondrial fractions, correspondingly. IOD of cytochrome C (B), Bax (C), and Bid (D) was determined with loading control normalization for each lane. Results in B, C, and D illustrate the mean ± SEM of 3 independent experiments. The * represents statistical difference as compared to control group, respectively (*p* < 0.05).

Previous studies have shown Bcl‐2 family proteins participated in the intrinsic apoptosis pathway and the regulation of mitochondrial integrity.^12^, ^41^ To determine the relationship between intrinsic mitochondrial pathway with Bcl‐2 family proteins of arsenic‐induced apoptosis in MA‐10 cells, expression of Bid, Bax, and cytochrome C proteins in mitochondrial and cytosolic fractions were investigated. Results showed that sodium arsenite at 10 μM for 24 h considerably stimulated cytochrome C release in dose‐dependent (Figure 2A,B) (*p* < 0.05) and time‐dependent manners (Figure 3A,B) (*p* < 0.05), correspondingly. In addition, sodium arsenite significantly induced mitochondrial over cytosolic Bax ratio in dose‐ and time‐dependent manners (Figure 2A,C, 3A,C) (*p* < 0.05), showing Bax translocation from cytosol to mitochondria. The Bid expression in cytosolic fraction was significantly decreased in a dose‐dependent pattern (Figure 2A, D) (*p* < 0.05) and a time‐dependent manner (Figure 3A,D) (*p* < 0.05), correspondingly. Moreover, 10 mM dimethylarsenic acid at 12 and 24 h significantly induced cytochrome C release and Bax translocation (Figure 4A–C) (*p* < 0.05); and at 1 mM induced cytochrome C release and Bax translocation in a time‐dependent pattern (Figure 5A–C) (*p* < 0.05). After dimethylarsenic acid treatments, cytosolic Bid expression was significantly decreased in dose‐ and time‐dependent pattern (Figure 4A,D,5A,D) (*p* < 0.05). These data highly indicate both sodium arsenite and dimethylarsenic acid could significantly affect the mitochondrial integrity by modulating Bcl‐2 family proteins to induce apoptosis in MA‐10 cells.

### 3.3 Effects of arsenic compounds on MAPK pathway (JNK, p38 and RK1/2) activation in MA‐10 cell apoptosis

**FIGURE 6:**
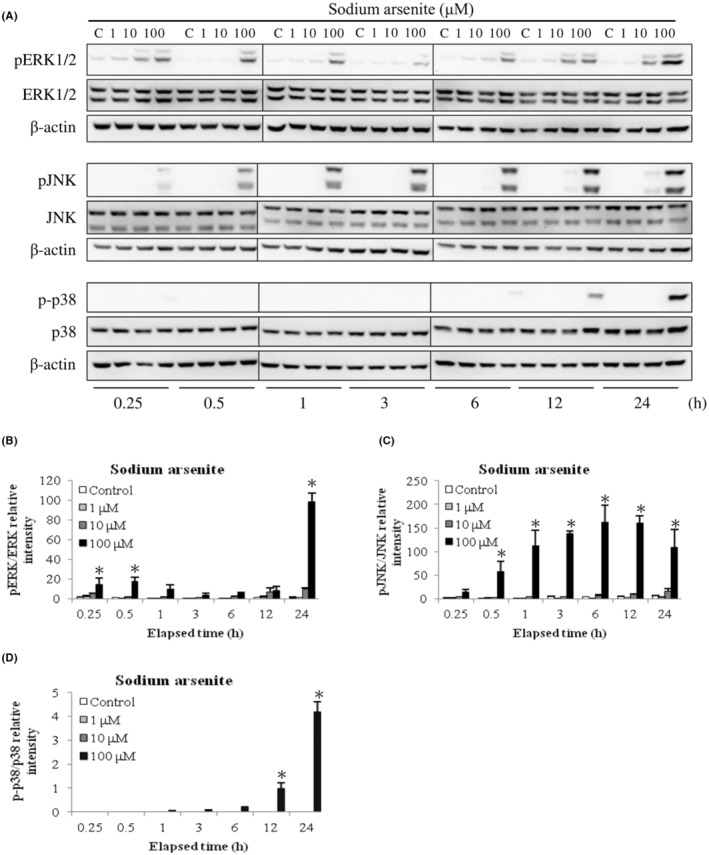
Sodium arsenite regulated phosphorylation of ERK1/2, JNK and p38 pathways in MA‐10 cells. Cells were challenged with sodium arsenite (0, 1, 10 and 100 μM) for 0.25, 0.5, 1, 3, 6, 12, and 24 h, respectively. Total and phosphorylated ERK1/2 (ERK1/2 and pERK1/2; 44/42 kDa), JNK (JNK and pJNK1/2; 54/46 kDa), and p38 (p38 and p‐p38; 43 kDa) proteins were detected via western blotting. Represent immunoblots are from one single experiment repeated at least 3 times (A). IOD of pERK1/2 (B), pJNK (C), and p‐p38 (D) proteins was determined after total ERK1/2, JNK and p38 normalization, respectively. Results in B, C, and D illustrate the mean ± SEM of 3 independent experiments. The * represents statistical difference as compared to control group, respectively (*p* < 0.05).

**FIGURE 7:**
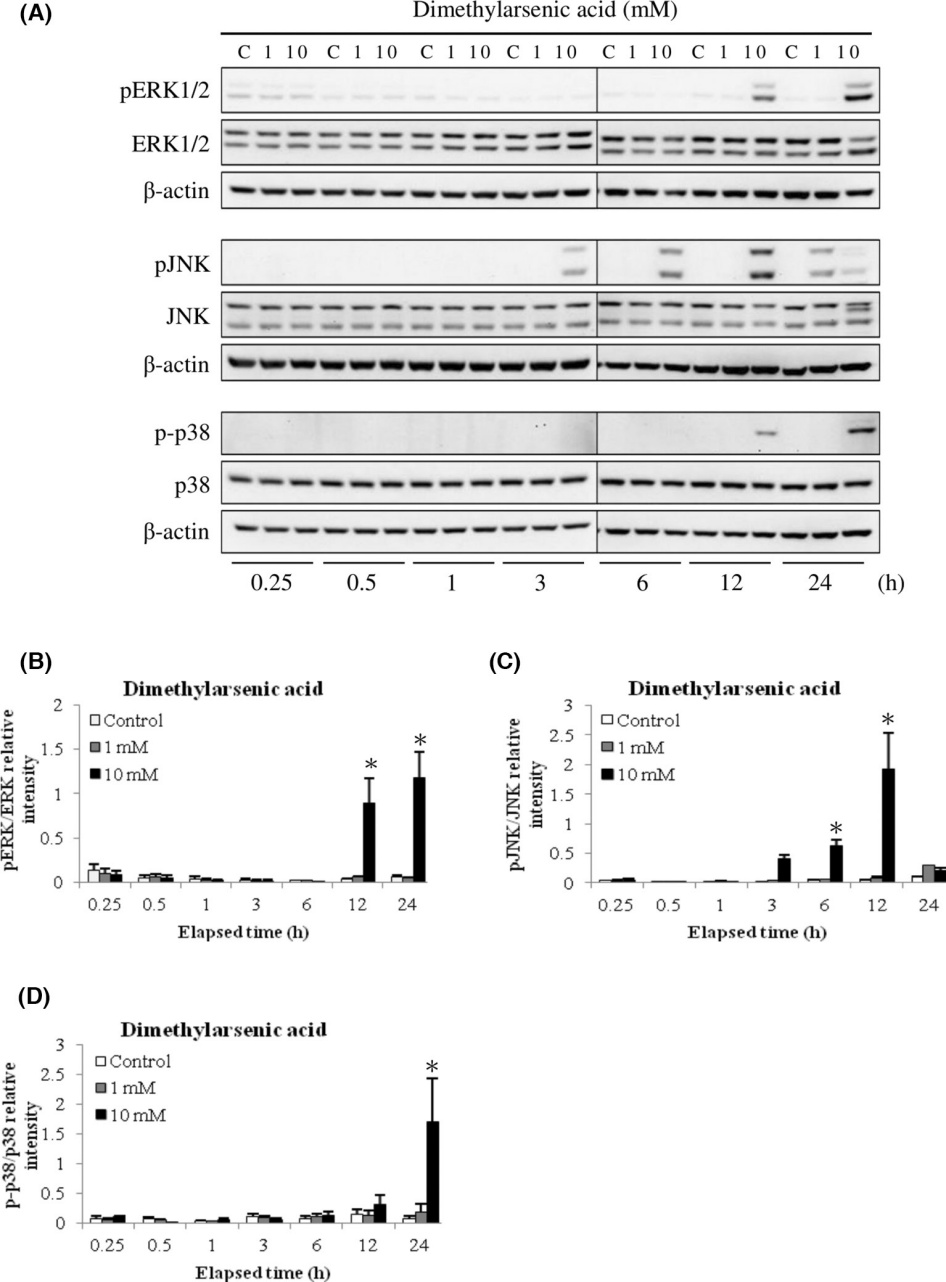
Dimethylarsenic acid regulated phosphorylation of ERK1/2, JNK and p38 pathways in MA‐10 cells. Cells were challenged with dimethylarsenic acid (0, 1 and 10 mM) for 0.25, 0.5, 1, 3, 6, 12, and 24 h, respectively. Total and phosphorylated ERK1/2 (ERK1/2 and pERK1/2; 44/42 kDa), JNK (JNK and pJNK1/2; 54/46 kDa), and p38 (p38 and p‐p38; 43 kDa) proteins were detected via western blotting. Represent immunoblots are from one single experiment repeated at least 3 times (A). IOD of pERK1/2 (B), pJNK (C), and p‐p38 (D) proteins was determined after total ERK1/2, JNK and p38 normalization, respectively. Results in B, C, and D illustrate the mean ± SEM of 3 independent experiments. The * represents statistical difference as compared to control group, respectively (*p* < 0.05).

It is well known that activation of MAPK pathway would negatively or positively regulate cell proliferation, mitosis and/or apoptosis.^22^, ^42^, ^43^ For determining whether arsenic‐induced apoptosis in MA‐10 cell could be facilitated through MAPK signaling pathway, activation of JNK, p38 and ERK1/2 in arsenic‐induced MA‐10 cell apoptosis were determined via western blotting. Data showed 100 μM sodium arsenite in 0.25, 0.5 and 24 h treatments significantly stimulated ERK1/2 phosphorylation (Figure 6A,B); in 0.5 to 24 h treatments considerably induced JNK phosphorylation (Figure 6A,C); and in 12 plus 24 h treatments considerably induced p38 phosphorylation (Figure 6A,D), respectively (*p* < 0.05). In addition, 10 mM dimethylarsenic acid in 12 plus 24 h treatments profoundly induced ERK1/2 phosphorylation (Figure 7A,B); in 6 plus 12 h treatments significantly induced JNK phosphorylation (Figure 7A,C); and in 24 h treatment obviously stimulated p38 phosphorylation (Figure 7A,D), respectively (*p* < 0.05). Interestingly, arsenic compounds stimulated different MAPK pathways (JNK, p38 and/or ERK1/2) via different time scales. In sodium arsenite treatments, the two phases of ERK activation were detected by first wave at 0.25–1 h and the second wave at 6–24 h; one phase of JNK activation from 0.5 to 24 h; and followed by one phase of p38 activation at 12 and 24 h (Figure 6A–D). Different to sodium arsenite, dimethylarsenic acid firstly induced JNK activation from 3 to 24 h; and followed by ERK and p38 activations at 12 and 24 h, correspondingly (Figure 7A–D). These results indicated that arsenic compounds activated JNK, p38 and/or ERK1/2 MAPK pathways during MA‐10 cell apoptosis.

### 3.4 Effects of arsenic compounds on Akt pathway regulation in MA‐10 cell apoptosis

**FIGURE 8:**
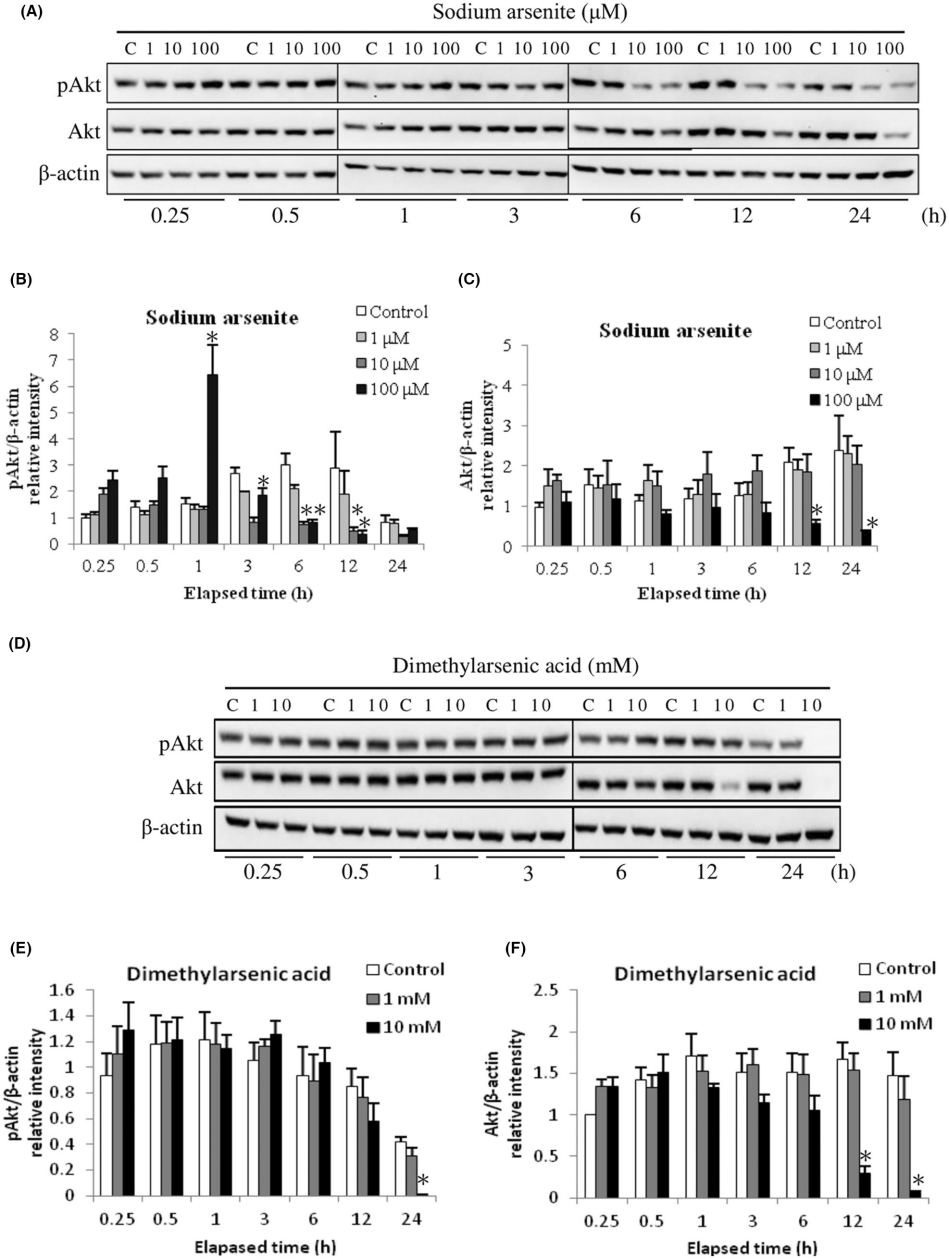
Sodium arsenite and dimethylarsenic acid regulated phosphorylation of Akt pathway in MA‐10 cells. Cells were challenged with sodium arsenite (0, 1, 10 and 100 μM) and dimethylarsenic acid (0, 1 and 10 mM) for 0.25, 0.5, 1, 3, 6, 12, and 24 h, respectively. Phosphorylated and total Akt (pAkt and Akt; 60 kDa) proteins were detected by western blotting. Represent immunoblots are from one single experiment repeated at least 3 times (A for sodium arsenite treatment and D for dimethylarsenic acid treatment). IOD of pAkt (B for sodium arsenite treatment and E for dimethylarsenic acid treatment) and Akt (C for sodium arsenite treatment and F for dimethylarsenic acid treatment) proteins was determined after β‐Actin normalization. Results in B, C, E, and F illustrate the mean ± SEM of 3 independent experiments. The * represents statistical difference as compared to control group, respectively (*p* < 0.05).

Study has shown Akt protein is an important pathway for cell survival, which closely relates to MAPK pathways.^44^ To investigate whether Akt signaling pathway might participate in MA‐10 cell apoptosis induced by arsenic compounds, phosphorylated and total Akt protein expressions were determined via western blotting. Results demonstrated 10 and 100 μM sodium arsenite significantly reduced Akt phosphorylation at 6 and 12 h (Figure 8A,B) (*p* < 0.05). Moreover, total Akt protein expression was profoundly suppressed after 12 and 24 h treatments by 100 μM sodium arsenite (Figure 8A,C) (*p* < 0.05). Similar to sodium arsenite, 10 mM dimethylarsenic acid significantly reduced Akt phosphorylation in 24 h treatment (Figure 8D and E) and total Akt protein expression in 12 and 24 h treatments (Figure 8D and F), respectively (*p* < 0.05). These results showed arsenic compounds would decrease Akt phosphorylated levels and Akt expression inhibiting survival signaling pathway in MA‐10 cells.

### 3.5 Effects of arsenic compounds on ROS generation in MA‐10 cell apoptosis

**FIGURE 9:**
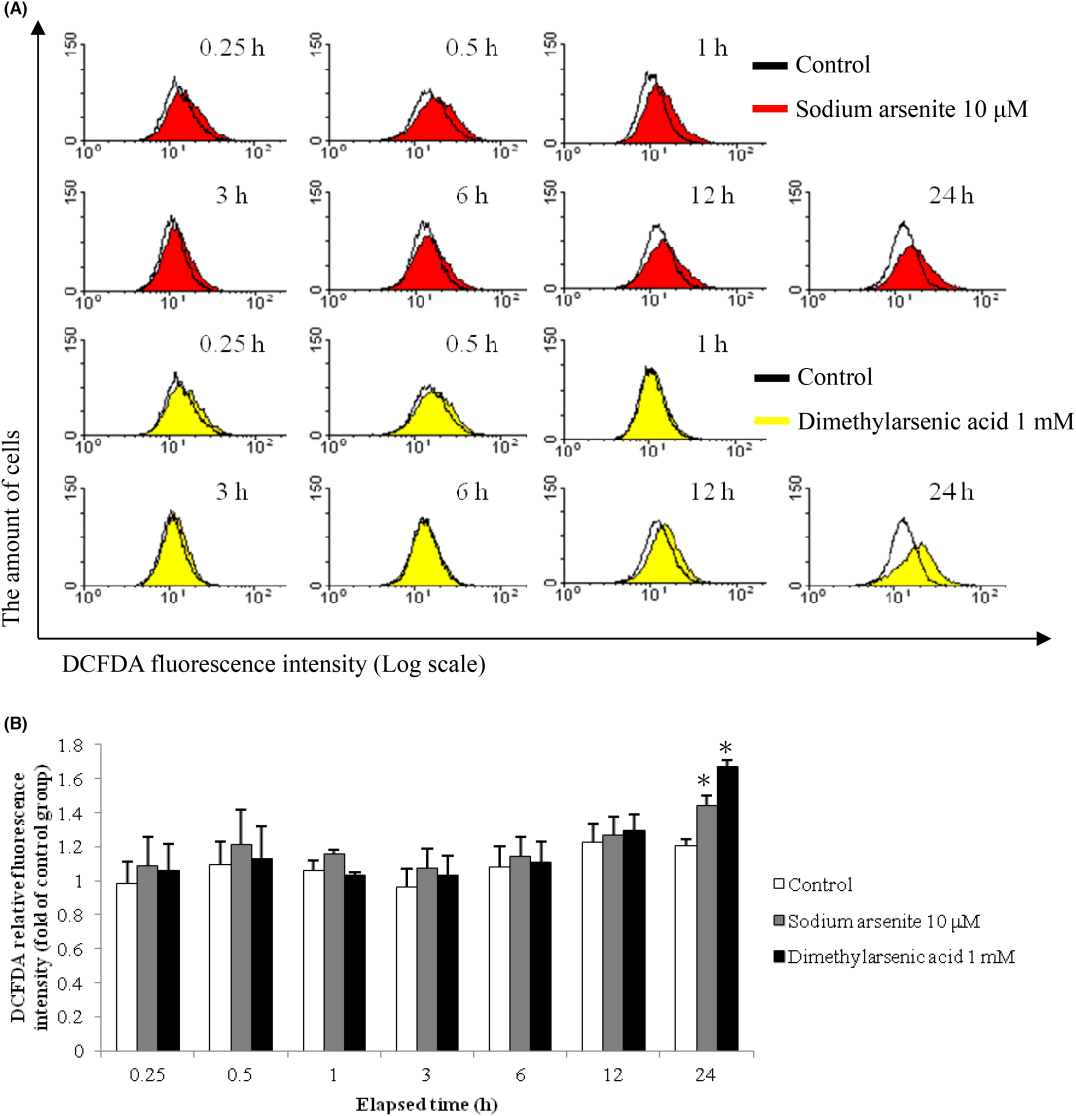
Sodium arsenite and dimethylarsenic acid modulated ROS generation in MA‐10 cells. MA‐10 cells were challenged with sodium arsenite (0 and 10 μM) or dimethylarsenic acid (0 and 1 mM) for 0.25, 0.5, 1, 3, 6, 12, and 24 h, respectively, and stained with DCFDA for 5 min prior to treatment time. Fluorescence was examined by flow cytometry and histograms represent DCFDA fluorescence intensity on a log scale (A). The quantification of ROS fluorescence intensity was normalized by control groups (B). Data in B represent mean ± SEM of 3 independent experiments. The * and ** represent statistical difference as compared to control group at *p* < 0.05 and *p* < 0.01, respectively.

Studies have shown excessive ROS generation could result in dysfunction of mitochondria and eventually led to cell apoptosis.^45^ To determine whether ROS might be involved in MA‐10 cell apoptosis by arsenic compounds, ROS generation was monitored via DCFDA staining and flow analysis among different treatments. Results showed that DCFDA fluorescence peaks shifted to the right on the X axis in the histograms (Figure 9A), which reflected intracellular ROS elevation. The intracellular ROS elevation was significantly stimulated after 24 h treatment by 10 μM sodium arsenite plus 1 mM dimethylarsenic acid, and dimethylarsenic acid induced greater ROS generation than sodium arsenite (Figure 9B) (*p* < 0.05).

**FIGURE 10:**
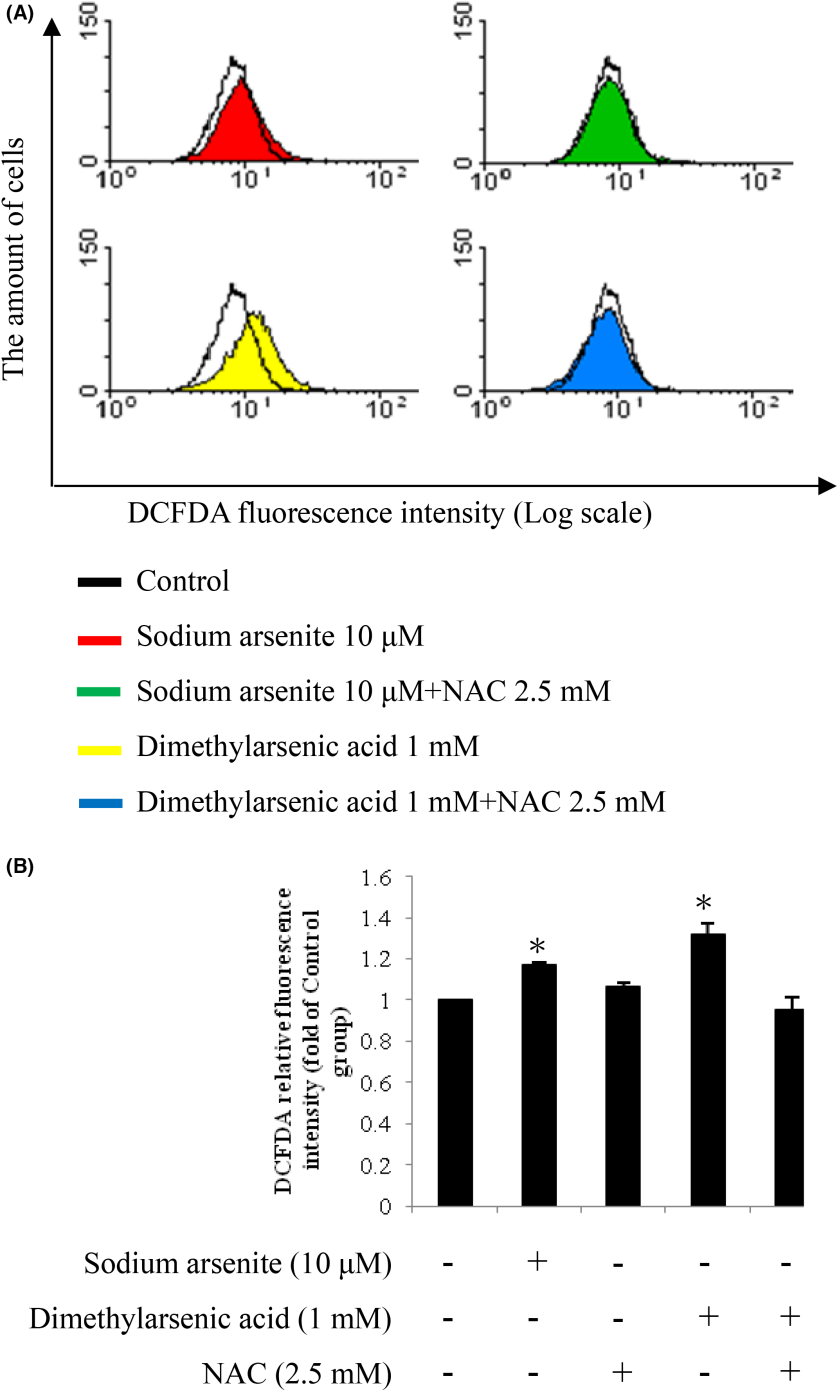
Effects of N‐acetyl‐L‐cysteine (NAC) on sodium arsenic‐ and dimethylarsenic acid‐induced ROS generation in MA‐10 cells. Cells were pretreated with NAC (2.5 mM) for 2 h and cotreated with sodium arsenite (0 and 10 μM) or dimethylarsenic acid (0 and 1 mM) for 24 h more, respectively. MA‐10 cells were stained with DCFDA for 5 min before treatment time. Fluorescence was examined by flow cytometry and histograms represent DCFDA fluorescence intensity on a log scale (A). The quantification of ROS fluorescence intensity was normalized by control groups (B). Data in B represent mean ± SEM of 3 independent experiments. The * and ** represent statistical difference as compared to control group at *p* < 0.05 and *p* < 0.01, respectively.

ROS scavenger, N‐acetyl‐L‐cysteine (NAC), was used to further explore whether arsenic compounds could induce MA‐10 cell apoptosis through intracellular ROS generation. Thus, 2.5 mM NAC was used to pretreated MA‐10 cells for 2 h and then with 10 μM sodium arsenite or 1 mM dimethylarsenic acid treatments for 24 h more, respectively. Histograms showed a leftward shift in NAC pretreatment groups, illustrating that arsenic‐induced ROS generation was inhibited (Figure 10A). The statistical analysis further demonstrated intracellular ROS level was reduced by NAC (Figure 10B) (*p* < 0.05), indicating sodium arsenite plus dimethylarsenic acid could stimulate intracellular ROS generation to induce apoptosis in MA‐10 cells.

## 4 DISCUSSION

We previously observed sodium arsenite or dimethylarsenic acid could induce apoptosis in MA‐10 cell through caspase‐8 and caspase‐9 apoptotic pathway, respectively.^3^, ^5^, ^7^ To additional determine role of intrinsic pathway, current results demonstrated the expression of FasL only did significantly increase after treatment of sodium arsenite. It was suggested that sodium arsenite or dimethylarsenic acid played different roles upon apoptosis in MA‐10 cells. It was also found that some of anticancer drugs, such as cisplatin and doxorubicin, induced Fas clustering in a FasL‐independent fashion and recruited FADD for activating caspase‐8 pathway and then cell apoptosis.^46^, ^47^ According to our findings, it could be suggested that dimethylarsenic acid‐induced cell apoptosis might be mediated with FasL‐independent Fas pathway to trigger caspase‐8 activation, and sodium arsenite‐induced cell apoptosis might be mediated with FasL‐dependent Fas pathway instead.

It is well known that mitochondrial integrity can be maintained through Bcl‐2 family proteins. In apoptosis, activation of Bax and Bak proapoptotic Bcl‐2 proteins is related to oligomerization and insertion upon outer mitochondrial membranes disturbing mitochondrial functions.^16^ Bax translocation subsequently increases mitochondrial permeability with cytochrome C efflux.^12^, ^15^ It has been reported that arsenic trioxide could inhibit functions of mitochondria of MCF7 breast cancer cells.^48^ Arsenic trioxide could also disrupt mitochondrial membrane potential to sensitize radiation therapy in mouse lung carcinoma model.^49^ Our results demonstrated that dimethylarsenic acid and sodium arsenite induced truncation of Bid, translocation of Bax, and release of cytochrome C, which highly indicated that arsenic compounds did disturb mitochondrial function through Bax translocation in MA‐10 cells. Moreover, Bid proteins, which were considered as the bridge between intrinsic and extrinsic pathways, can be cleaved into truncated Bid (tBid) by activated capase‐8, and tBid consequently enhances the mitochondrial permeability to facilitate cell apoptosis.^50^ Thus, Bid truncation in arsenic‐induced MA‐10 cell apoptosis could possibly explain the induction of both intrinsic and extrinsic apoptotic pathways. Cytochrome C release also reemphasized intrinsic pathway participation in MA‐10 cell apoptosis affected by arsenic compounds.

MAPK pathway is an essential regulatory pathway, including JNK, ERK1/2 and p38 MAPK,^43^ which has been illustrated the activation of JNK and p38 pathways could directly induce mitochondrial apoptosis pathway.^22^, ^51^, ^52^ Current data showed 10 and 100 μM sodium arsenite and 1 and 10 mM dimethylarsenic acid, respectively, induced JNK phosphorylation during caspase activation. It has been shown that JNK can induce Bax translocation and trigger mitochondrial cell apoptosis.^53^ Thus, our data highly indicated that JNK phosphorylation could participate in MA‐10 cell apoptosis induced by arsenic compounds. Moreover, the phosphorylation of p38 MAPK was found after treatment with sodium arsenite (100 μM) and dimethylarsenic acid (10 mM) in this study. Many evidences have revealed p38 protein acts as a cell cycle regulator to direct cell survival and death.^54^ Thus, our observations regarding p38 protein role in MA‐10 cell apoptosis induced by arsenic compounds are comparable. In addition, study has shown sustained and transient activations of ERK have different effects on cell growth stimulation and inhibition through ERK nuclear translocation in human hepatoma cells.^55^ Furthermore, sustained phosphorylation of ERK via constitutively active MEK would induce apoptosis without any treatments.^56^ In our results, ERK activation was induced by 10 and 100 μM sodium arsenite and 10 mM dimethylarsenic acid, respectively, in 12 and 24 h treatments along with significant caspase activation, consisting with human mesothelioma cell study.^23^ Our results indicated that arsenic compounds might induce late phase ERK phosphorylation, and ERK activity was closely related to MA‐10 cell apoptosis induced by arsenic compounds.

PI3K/Akt signaling could be activated through various growth factor receptors regulating cell proliferation, survival and growth. Also, Akt signaling would disturb proapoptotic ability of caspase‐9 and Bad proteins.^25^, ^57^ The dysregulation of Akt signaling could be observed in some cancers through either constitutive active growth signals or overexpression of PI3K and Akt proteins.^58^ In our results, the phosphorylation of Akt was gradually decreased after treatment with sodium arsenite and dimethylarsenic acid. It is well known that inactive Akt is unable to suppress apoptosis, and our data showed that caspase cascade was subsequently induced after 12 h arsenic treatments in MA‐10 cells. Moreover, the level of total Akt protein decreased in high dose of dimethylarsenic acid and sodium arsenite, and it has been reported arsenic trioxide decreased Akt protein expression via caspase‐dependent pattern in leukemic cells.^59^ Thus, our observations suggested that Akt inactivation might facilitate arsenic‐induced MA‐10 cell apoptosis.

It has been shown that excessive ROS generation would induce intrinsic and extrinsic pathways to stimulate cell apoptosis.^60^, ^61^ Also, it has been shown arsenic trioxide could induce ROS elevation to stimulate apoptosis in HONE‐1 nasopharyngeal carcinoma cells.^62^ Moreover, dimethylarsenic acid could induce cell apoptosis through glutathione redox system, which was closely related to ROS homeostasis in TRL 1215 rat epithelial liver cells.^63^ Consistent with those studies, our results showed that both sodium arsenite and dimethylarsenic acid induced ROS generation, which was eliminated by pretreatment with ROS scavenger, NAC. Moreover, it is illustrated that prolonged ROS production participated in arsenic‐induced JNK activation in NF‐κB deficiency mice model.^64^ According to JNK activation in MA‐10 cells, it was suggested that ROS production might be associated with JNK activation in MA‐10 cell apoptosis induced by arsenic compounds.

Taken together, arsenic compounds could induced MA‐10 cell apoptosis by activating extrinsic and intrinsic cascades of caspase pathways with Bid truncation, cytochrome C release and Bax translocation, plus excessive ROS generation. Furthermore, sodium arsenite, but not dimethylarsenic acid, participated in Fas/FasL pathway. Moreover, arsenic‐induced cell apoptosis could be mediated by MAPK pathway up‐regulation of and Akt pathway suppression in MA‐10 cells.

Arsenic trioxide was approved for an anti‐tumor drug to treat acute promyelocytic leukemia.^9^, ^10^ Moreover, studies have demonstrated that arsenic trioxide, dimethylarsenic acid and/or phenylarsonous acid could induce cell death and apoptosis in prostate cancer, ovarian cancer, TM4 Sertoli tumor cells, and oral cancers plus clinical investigations related to cancer therapy.^3^, ^4^, ^5^, ^6^, ^7^, ^8^ *Thus, our observations upon apoptotic effect of* arsenic compounds *in MA‐10 mouse Leydig tumor cells could highpoint the potential anti‐tumor therapeutic issue for clinical application related to tumors in reproductive systems*.

## AUTHOR CONTRIBUTIONS

Present study was designed by WSJ, YFM, SCL and BMH. Experiments were performed by WSJ, YFM, ECS and YPL. Raw data authenticity was confirmed by WSJ, CYW, SCL and BMH. Data were analyzed by WSJ, YFM, SCL and BMH. Results were interpreted by YFM, CYW and YPL. Initial manuscript was drafted by WSJ, YFM, ECS and YPL. Manuscript for important intellectual content was revised by WSJ, CYW, SCL and BMH. Final manuscript was read and approved ensuring accuracy or integrity of the work by all authors.

## FUNDING INFORMATION

Present study was supported by Ministry of Science and Technology grants MOST110‐2320‐B‐006‐025‐MY3 (BMH) and the An Nan Hospital grant ANHRF110‐36 (WSJ and BMH), Taiwan, Republic of China.

## CONFLICTS OF INTEREST

Authors declare no conflict of interest and no competing interests.

## ETHICS APPROVAL AND CONSENT TO PARTICIPATE

Not applicable.

## Data Availability

Data used and analyzed of this study are always accessible through authors on reasonable demand.
